# Examining Practices Related to Ethical Aspects in eHealth Evaluation Research: Protocol for a Scoping Review

**DOI:** 10.2196/60849

**Published:** 2025-05-05

**Authors:** Linda Wienands, Sabine Valenta, Lynn Leppla, Sabina De Geest, László Kovács, Alexandra Teynor, Julia Krumme

**Affiliations:** 1 Department of Medicine Medical Center – University of Freiburg, Faculty of Medicine University of Freiburg Freiburg Germany; 2 Institute of Nursing Science Department Public Health University of Basel Basel Switzerland; 3 Practice Development and Research Division Medical Directorate University Hospital Basel Basel Switzerland; 4 Nursing Direction Medical Center – University of Freiburg, Faculty of Medicine University of Freiburg Freiburg Germany; 5 Academic Centre for Nursing and Midwifery Department of Public Health and Primary Care KU Leuven Leuven Belgium; 6 Faculty of Liberal Arts and Sciences Technical University of Applied Sciences Augsburg Augsburg Germany; 7 Faculty of Computer Science Technical University of Applied Sciences Augsburg Augsburg Germany

**Keywords:** cancer, cardiovascular, eHealth, remote patient monitoring, ethics, ethical aspects, applied ethics, evaluation designs, evaluation research, scoping review, protocol

## Abstract

**Background:**

eHealth technologies, including remote patient monitoring (RPM) applications, have the potential to improve care for diseases such as cancer and cardiovascular conditions. However, they also raise ethical aspects that are often inadequately addressed in eHealth evaluation research. This is problematic, as evaluations guide decision-making at multiple levels. To improve evaluation practices, it is essential to understand how ethical aspects are addressed in terms of both content and methodology, enabling the development of tailored recommendations for enhancement.

**Objective:**

This scoping review systematically examines how ethical aspects are addressed in eHealth research, focusing on original studies evaluating RPM applications for cancer and cardiovascular diseases.

**Methods:**

Using Joanna Briggs Institute (JBI) methodology and PRISMA-ScR (Preferred Reporting Items for Systematic Reviews and Meta-Analyses extension for Scoping Reviews) guidelines, this review implemented a comprehensive search strategy with the terms “cancer or cardiovascular diseases,” “eHealth or telemonitoring,” and “evaluation designs.” Searches included MEDLINE, Embase, CINAHL, SocINDEX, Philosopher’s Index, PsycINFO, and Google Scholar. Data extraction will emphasize ethical aspects and methodological approaches to consider them. The analysis will apply inductive-deductive qualitative content analysis.

**Results:**

Initial searches identified 3321 articles published between 2014 and August 2024. Screening and analysis will be completed in the first quarter of 2025, with results anticipated by summer 2025.

**Conclusions:**

Overlooking ethical aspects in evaluation studies can significantly impact eHealth practices. This scoping review will map ethical considerations in original evaluation research, identifying opportunities for more holistic integration of ethics and informing future practical guidance.

**Trial Registration:**

OSF Registries OSF.IO/7XAFV; https://osf.io/7xafv/

**International Registered Report Identifier (IRRID):**

DERR1-10.2196/60849

## Introduction

### Background

Technologies have significantly transformed health care delivery in recent decades. Remote patient monitoring (RPM) pertaining to eHealth exemplifies this transformation by enabling health care services to extend beyond traditional hospital settings. RPM addresses many limitations of clinical environments, such as dependency on physical infrastructure, and reduces geographical barriers, thereby enhancing care accessibility for diverse populations [1]. RPM leverages tools, such as smartphones, web-based platforms, wearable devices, and biomedical sensors, to collect health-related data, including vital signs and symptoms, directly from patients. These data are transmitted to health care providers, enabling them to monitor patients’ health progress and promptly intervene in cases of deterioration [2]. RPM shows particular promise in the care of cancer [3,4] and cardiovascular diseases [5], as these conditions often require prompt intervention when health issues arise. Together, they account for more than half of global mortality, with 33% attributed to cancer and 18% to cardiovascular diseases [6]. By enabling early detection of health issues and interventions, such as adjusted medical regimens or prompted medical visits, RPM has the potential to improve the quality of life and even save the lives of patients managing these chronic conditions [7-10].

With the growing adoption of eHealth technologies, such as RPM, the demand for empirical evidence has risen. Evaluation research aims to enhance practical application, generate knowledge, and support informed decision-making regarding an evaluation objective [11]. Ideally, such studies accompany the entire eHealth life cycle, encompassing stages from preprototype and prototype development to pilot testing, demonstration, scaling, and sustained implementation [12,13]. For instance, considering clinicians’ preferences at the pre-prototype stage helps tailor eHealth solutions to users’ needs, whereas testing prototypes ensures usability and identifies technical issues. Such studies also assess impacts such as improved patient quality of life and uncover factors for successful implementation [14].

There is growing consensus that empirical evaluations alone are insufficient to ensure that eHealth is ethically sound, which is an essential standard in health care [15-17]. This is reflected by recommendations from the World Health Organization (WHO), which emphasize the importance of integrating ethical considerations into eHealth practices, including those related to development, implementation, use, and research [1,18,19]. Despite these recommendations, issues such as unequal access to technologies and risks to data security have been insufficiently addressed in studies [17,20-26]. This is concerning because the findings of evaluation research directly impact the development of technologies, health care delivery, and policy decisions, placing significant responsibility on evaluators and researchers [27-29]. Neglecting ethical considerations in these studies, therefore, risks their insufficient representation in decision-making processes.

To promote ethical practices in eHealth, it is essential to address the limited attention paid to ethical aspects in evaluation research, which may stem from methodological challenges related to the application of ethics [30,31]. Interdisciplinary teams, often lacking ethicists, struggle not only to understand what ethical issues to consider but also how to address them. However, comprehensive guidance, particularly for original evaluation research, remains scarce [31]. To create such guidance, a comprehensive overview of eHealth evaluation research practices is needed to identify challenges and opportunities in addressing ethical aspects in this context [22]. This can inform practice recommendations for researchers that are better aligned with and adaptable to existing evaluation practices. However, the field remains underexplored.

The complexity of ethics, lack of targeted guidance for interdisciplinary teams, and absence of comprehensive reviews of evaluation research practices provide the rationale for this scoping review. These issues are explored in detail in the following sections and further elaborated in the proposed review, using evaluations of RPM in cancer and cardiovascular care as practical case studies.

### The Broad Spectrum of Ethical Aspects in eHealth Evaluation Research

The ethical aspects in the literature include questions, concerns, risks, issues, challenges, fallacies, and considerations. Although no universal definition of these aspects exists, there is consensus among proposed definitions regarding their normative nature, as they include implicit or explicit claims about how individuals should behave, act, or how specific circumstances ought to be [32] (eg, the topic “health disparities” implies that access to health care ought to be fair).

In the context of eHealth, ethical aspects concern the impact of technologies on individuals and society. They also relate to the responsibilities and actions of professionals in areas such as software development, health care, and research [33]. Thus, although not exhaustive, 2 key categories—the process domain and the outcome domain—are particularly relevant for classifying ethical aspects in eHealth evaluation research [34,35].

First, the ethical aspects of the process domain: Evaluation research is closely linked to value judgments, which unavoidably connect the process to ethical aspects. Ethical aspects are especially evident when the distribution of power between interest groups is unequal [30]. Kelly et al [36] highlighted several ethical concerns in the eHealth field. Decisions about a study’s focus, design, and conclusions are often shaped by the values of individual stakeholders. For instance, stakeholders in influential positions, such as members of the pharmaceutical industry, tend to exert a greater influence on decision-making processes. They may determine which health technologies receive funding and are prioritized for research, with choices frequently guided by economic interests. While this profit-driven perspective can foster innovation, it does not always align with actual health care needs, potentially resulting in missed opportunities for improving patient care. The focus on profit can clash with goals such as fairness and justice, which emphasize benefits for society as a whole.

Second, the ethical aspects on the outcome domain: Ethical aspects of eHealth are linked to unintended and dual outcomes arising from the interactions between the intervention components and the environment in which it is applied [37]. An example in this domain is that, while eHealth aims to improve health care access for rural populations, concerns arise about worsening health disparities [38-40]. Disadvantaged groups, such as individuals with lower educational levels or socioeconomic status, may not benefit from eHealth as intended. These patient groups were frequently excluded from the studies, creating a problematic selection bias. If this ethical concern is neglected and eHealth methods are not adapted, health disparities will likely increase [38-41]. In this regard, the underreporting of negative effects associated with eHealth is of particular concern, which impedes a comprehensive understanding of these unintended and dual effects [42].

In summary, the ethical aspects discussed in the literature are diverse and context-specific, emerging from systematic reflections within the broad eHealth field. This makes them less ready to be operationalized and investigated compared with empirical outcomes such as effectiveness. Without proper guidance, this poses a challenge for non-ethicists involved in eHealth evaluations and may contribute to the limited focus on ethical aspects [31].

### Integrating Ethical Analysis Into eHealth Evaluation Research

Guidelines for evaluating health technologies, such as those from the WHO [12], lack explicit instructions on how to incorporate ethical considerations into empirical evaluation designs. This reflects the traditional separation between empirical and ethical approaches, which foresees that ethical aspects are considered in systematic ethical analyses as supplementary research [43]. While such systematic analysis can take various forms, it typically includes key components [44]. Simplified, these entail a descriptive assessment of the current situation (the “Is” aspect) and a critical reflection on ethically desirable outcomes guided through ethical approaches (the “Ought” aspect). Among others, these approaches may include the application of overarching moral theories such as consequentialism, deontology, and virtue ethics [45], or context-specific principles from applied ethics [32]. Finally, an ethical analysis entails the formulation of ethically justified recommendations for practice (the “Action” aspect) [44].

The application of ethics in the context of eHealth is an evolving field [37]. The European VALIDATE (VALues In Doing Assessments of health Technologies) project [43] is one initiative focused on developing guidance for the Health Technology Assessment (HTA) context—a process used to evaluate the value of health technologies at various life cycle stages [46]. The findings from the project emphasize that rigid separations between empirical and ethical approaches are problematic. This is because ethical aspects are integral to the empirical evaluation process itself [43], as outlined above, in connection with the ethical aspects of the process and outcome domain. Therefore, it is recommended to systematically integrate ethical considerations into evaluation research practices, rather than treating them in isolation. The VALIDATE handbook offers guidance on integrated approaches for the HTA process, which aims to create reports based on existing data. Thus, it can serve as an initial orientation for the evaluators. However, the handbook does not adequately address the inclusion of ethical considerations in original evaluation research, where the collection of new evaluation data poses specific ethical challenges that require tailored guidance [47].

Integrating ethics into original evaluation research can offer significant benefits, both in terms of addressing ethical aspects during the process and in the outcome domain:

First, the ethical aspects of the process domain: Incorporating ethical approaches into evaluation research encourages reflection on the underlying values and power dynamics. By addressing the ethical challenges identified during this reflection through proposing or applying corrective measures, researchers can enhance the ethical integrity of both the evaluation process and its objective, namely the eHealth technology [36,48].

Ethical aspects in the research process domain are relevant throughout all stages of the eHealth life cycle, regardless of the evaluation design or methods. This is evident in the practice of submitting study protocols to ethics committees, which promotes ethical reflections on methodological choices. However, this approach is limited because it primarily occurs during the study conceptualization phase, varies across countries, and lacks consistent quality standards among ethics committees [47,49].

Beyond the requirement for ethics approval, reporting guidelines and scientific journals encourage authors to address process-related ethical aspects in their final publications. This may involve reflections on participant selection and its impact on result interpretation [27], which is typically discussed in the methods and discussion sections. Another opportunity for including ethical considerations in manuscripts lies in the author’s statements, which can encompass reflections on funding influences and value-based decisions, often reported under conflicts of interest [50].

Second, the ethical aspects on the outcome domain: Integrating ethical considerations into the outcome domain of original evaluation research can improve the focus on unintended and dual effects of eHealth interventions.

For instance, ethical reflections at early stages, such as conceptualization and design, help identify potential ethical issues and proactively address them [51]. Furthermore, integrating ethics throughout the evaluation life cycle ensures continuous assessment from the initial stages to long-term effectiveness studies [52]. Grounding ethical reflections in current evidence leads to practical and relevant recommendations rather than purely theoretical ones [53]. As evaluation research is closely linked to real-world eHealth projects, ethical considerations can be applied immediately [54], supporting ethical informed development, implementation, and long-term use. Ultimately, integrating ethics into evaluation research results in well-founded, justified recommendations that are more likely to be accepted by various stakeholders and effectively implemented in practice [32,55].

Qualitative studies are particularly suitable for uncovering unintended or dual effects, owing to their exploratory nature and necessary reflexivity [53,56,57]. Although less detailed, quantitative studies are also intended to address ethical aspects. For instance, the CONSORT (Consolidated Standards of Reporting Trials) eHealth guideline suggests that randomized controlled trials transparently report unintended effects, such as harm or privacy concerns, in final reports [58].

To sum up, existing evaluation research practices already encourage ethical reflections, albeit often unconsciously and in a less systematic manner. Nevertheless, these practices provide a valuable foundation for developing more comprehensive guidance to enhance evaluation research and to move toward fully integrated approaches.

### Previous Work

To our knowledge, no review has examined in sufficient depth whether and to what extent ethical aspects are considered in original evaluation research. Some identified reviews relied on the search term “ethics” [22,23,59,60]. Through this, studies that addressed ethical aspects without explicitly labeling them were excluded [22]. Consequently, these reviews lack the necessary evidence to definitively conclude that ethical aspects are overlooked. For instance, a feasibility study by Appleyard et al [61] explored the barriers to eHealth adoption among older patients with cancer. This study highlighted the risk of digital exclusion and recommended tailoring eHealth systems to meet the needs of individuals with limited digital skills. Although the findings were not explicitly framed as ethical considerations, they inherently addressed critical ethical issues such as health disparities, the digital divide, and equity. Misclassifying such studies as neglecting ethical aspects risks reinforcing the misperception of ethics as a detached and isolated concern, rather than an integral part of evaluation research practices [43,62]. Therefore, a more thorough examination of the existing empirical practices related to ethical aspects is required.

Other reviews have focused on HTA reports [26,63,64] without exploring practices within original research studies. Reviews examining original research primarily focused on ethical aspects substantively [23,24,60,65,66], while largely overlooking the methodological domain—specifically, how ethical aspects were addressed (eg, which ethical approaches were used). Without considering these methods, they fail to capture whether ethical issues were considered appropriately. For example, some studies may only reference ethical concerns descriptively, like “eHealth can increase health disparities,” without offering a reflective analysis or actionable steps. A more comprehensive approach to eHealth would go beyond merely describing referenced issues; it would involve identifying, reflecting on, and proposing well-justified, practical solutions [44].

Only 1 review by Steerling et al [25] considered referenced ethical approaches in conjunction with reported ethical aspects. Nonetheless, the extent and systematic integration of ethical considerations in the included studies remain unclear. For instance, the review does not discuss the topic in connection with the studies’ evaluation design and eHealth life cycle stage, whether ethical aspects were addressed in the process or outcome domain, reported in the introduction or discussion section, and other essential details. These nuances are pivotal in mapping evaluation practices and making tailored suggestions for improvement.

As a consequence, the insights provided in previous reviews are insufficient to provide essential information for the development of guidance regarding integrated ethical approaches in original eHealth evaluation research. Given these limitations, a scoping review is an appropriate method for addressing this research gap. Scoping reviews are specifically designed to explore broad research questions, investigate unknown areas, map research practices, and offer conceptual clarification [67]. These characteristics align with the objectives of this review.

### Objective

Original evaluation research has great potential to promote ethical practices in eHealth by informing decision-making in this field. Therefore, addressing the current lack of focus on ethical aspects is crucial. A thorough examination of the existing evaluation research practices, which is the main goal of this scoping review, is an important first step toward improving this area.

The focus will be on studies examining bidirectional RPM technologies—those featuring interfaces for both patients and clinicians—within the context of cardiovascular and cancer care as practical case studies. The insights gained from this review will serve as a foundation for guiding empirical ethical evaluation research in the eHealth context for future studies.

### Review Questions

The review questions are as follows:

1. Are ethical aspects of eHealth implicitly or explicitly addressed in original eHealth evaluation research? (Ethical aspects—general overview)
2. Which ethical aspects (eg, process or outcome domain) are addressed? (Ethical aspects—classification)
3. To what extent are ethical aspects addressed in terms of ethical analysis steps and approaches? (Ethical approach—methodology)
4. What are promising starting points for implementing ethical approaches in original eHealth evaluation studies? (Potential for improvement)

## Methods

### Study Design and Review Team

The proposed scoping review will be conducted according to the Joanna Briggs Institute (JBI) scoping review methodology [68] and will follow the PRISMA-ScR (Preferred Reporting Items for Systematic Reviews and Meta-Analyses extension for Scoping Reviews; see Multimedia Appendix 1 for the PRISMA-ScR checklist) [69]. The review process is supported by Rayyan, an AI-powered web application for the creation of systematic reviews [70].

The interdisciplinary review team consists of nursing scientists (LW, SV, LL, and SDG), ethicists (JK and LK), and a software engineer (AT), bringing together empirical and ethical expertise. Within their respective disciplines, all review team members have a minimum of 3 years of experience with eHealth. To ensure consistency with the JBI Manual, the first author and main reviewer (LW) attended the JBI scoping review workshop in June 2022.

### Inclusion Criteria

#### Participants

This scoping review will consider studies that include clients and providers of eHealth services. The clients will include patients of any age who have cardiovascular disease or cancer. We will also consider studies that include individuals acting on behalf of patients (eg, parents and caregivers) who are involved in the collection of health data and the use of eHealth. Health care providers will be included if they use the collected information, for instance, by retrieving and monitoring it.

#### Concept

This review will focus on eHealth-enabling RPM. eHealth involves using “information and communications technologies in support of health and health-related fields” [1]. RPM is a health care delivery method that monitors patients’ health outside traditional clinical settings, using technology for information transmission between patients and health care providers [2]. RPM devices to be considered include noninvasive disease management applications and web-based platforms. This scoping review will exclude fully automated devices that operate without active patient participation in data entry or transmission, such as fitness trackers, cardiac implants, or medication dispensers. In addition, applications designed solely for patient use, such as those limited to sending notifications about health issues without involving communication with health care providers, will also be excluded.

#### Context

This review will consider studies investigating the use of eHealth in any clinical context (eg, hospital, outpatient clinic, specialist, or general practice) and in any geographical location. Studies evaluating the use of RPM in inpatient settings, such as intermediate care units or hospice care, will be excluded.

#### Types of Sources

This scoping review will include qualitative, quantitative, and mixed methods original research. Within the context of eHealth, evaluation studies may have diverse objectives, ranging from explorations of perceptions and experiences in qualitative designs to specific outcomes such as usability, acceptability, and costs in quantitative designs [12,14]. As such, no limitations for inclusion will be predefined in this regard. Studies during the conceptualization and development phases will be included only if testing of an eHealth prototype is reported. Secondary research, abstracts, methods, and opinion papers will be excluded from the proposed scoping review.

### Search Strategy

A comprehensive literature search was conducted in August 2024. The full search strategy used the keywords “cancer or cardiovascular diseases,” “eHealth or telemonitoring,” and “evaluation designs.” To create relevant search blocks, we conducted an initial limited search of MEDLINE (PubMed) to identify relevant articles. We used keywords from the titles, abstracts, and index terms of these articles to develop a full search strategy (Multimedia Appendix 2). The development process was supported by an information specialist from the University Library Basel (Switzerland) and involved previously published search blocks from the Biomedical Information study group of the Dutch Library Association as a reference [71].

The search strategy, which included all identified keywords and index terms, was modified for each information source. We accessed MEDLINE (PubMed), Embase (Ovid), PsycINFO (Ovid), CINAHL (EBSCOhost), SocINDEX (EBSCOhost), and Philosopher’s Index (EBSCOhost). Gray literature sources were searched through Google Scholar. To limit the influence of previous activities on Google, we searched in private browsing mode [72]. The search strategy focused on original studies published between 2014 and August 2024.

In accordance with the language skills of the main reviewers (LW and JK), articles published in English and German will be considered for screening.

### Source of Evidence Selection

After the search, all identified records were imported into the citation management software EndNote 20 (Clarivate Analytics) and subsequently transferred to the Rayyan platform. Duplicate entries were automatically removed using Rayyan when their similarity exceeded 95%. All remaining duplicates were manually reviewed.

To ensure consistency among reviewers, a pilot test of source selection will be conducted. Therefore, the main reviewers will screen 25 titles and abstracts to assess their eligibility based on the inclusion criteria. In line with recommendations, the independently selected articles will be compared, and the process will proceed when a consensus is reached on 75% of the selected articles [67].

Potentially relevant papers will be retrieved in full. The full texts of the selected citations will then be assessed by the main reviewers against the inclusion criteria. Reasons for excluding full-text papers will be recorded and reported in the scoping review. Any disagreements between the main reviewers at each stage of the selection process will be resolved through discussion. If consensus cannot be reached, advice from further reviewers will be sought, according to their field of expertise. The results of the search will be reported in full in the final scoping review and presented in a PRISMA (Preferred Reporting Items for Systematic Reviews and Meta-Analyses) flow diagram [69,73].

### Data Extraction

Data will be double-extracted from the papers included by the main reviewers (LW and JK). The reviewers will meet regularly during the process to cross-check each other’s extractions and discuss their findings. In case of disagreement between the two reviewers, further members of the review team will be consulted to reach consensus through discussion.

Basic study information will be extracted using the JBI data extraction instrument for scoping reviews [68]. In addition, studies will be screened for key findings relevant to the review questions 1-4 in two rounds.

Review questions 1 and 2: In the initial round, we will use the document search function to screen for the term “ethics” to identify aspects that are explicitly labeled. In the next step, we will thoroughly read the evidence sources, focusing on identifying ethical aspects within the full texts that are not labeled as such. Passages implying ethical aspects, such as critical reflections on decision-making in the research process or reported unintended effects, will be extracted. To aid identification, we will use the three moral theories: consequentialism, deontology, and virtue ethics. These theories offer universal guidance, covering a wider range of ethical aspects compared with context-specific principles [45] and have been previously utilized in another review to extract ethical aspects in the context of eHealth [25]. In addition, we will closely examine the authors’ use of normative language to express how things ought to be. This will be achieved by screening the text for modal auxiliaries such as “shall, should, ought to, must, need to.” Extracted passages indicating ethical aspects will be categorized into 2 domains: the process domain, pertaining to ethical aspects associated with the research process, and the outcome domain, concerning ethical aspects related to unintended effects of eHealth deployment.

Review question 3: In the second round, we will rescreen the studies with identified ethical aspects to extract methodological information. We will focus on text passages that clarify how authors address ethical aspects, including related descriptions (“Is” aspect), reasoning and justifications (“Ought” aspect), and recommendations (“Action” aspect).

Review question 4: In addition, we will take note of emerging ideas for the potential integration of ethical considerations and improved reporting of ethical aspects. Relevant text passages will be extracted for further analysis.

A draft extraction tool (Textbox 1) has been developed with preliminary categories “Ethical aspects” (with subcategories “Yes/No,” “Process domain,” “Output domain,” and “Manuscript section”), “Methodology” (with subcategories “Is,” “Ought,” and “Action”), and “Potential for improvement.” These categories for extracting key information to address the review questions will be further refined inductively during the data extraction and analysis processes. Any modifications to the extraction tool will be deliberated among the primary reviewers and will be documented in the final scoping review.

**Preliminary categories and subcategories of the draft extraction tool.**

- Ethical aspects:
  Yes/No
  Process domain
  Output domain
  Manuscript section
- Methodology:
  Is
  Ought
  Action
- Potential for improvement

### Data Analysis

To analyze the study information, we will conduct a descriptive statistical analysis to demonstrate the characteristics of the included studies [74]. The same applies to review question 1, where we will quantify the extent to which ethical aspects are addressed and present the findings in numbers and percentages.

To answer review questions 2-4, we will use a basic qualitative content analysis approach for scoping reviews [74]. We will apply a mixed deductive-inductive approach, following these steps: Data will be extracted deductively using the draft extraction tool. During the reading and initial extraction process, we will conduct open coding and note initial thoughts to further develop our extraction tool, also known as the coding framework. Once the coding framework is finalized, we will extract and organize relevant information within it. Throughout the process, we will review the coding framework and, if necessary, develop new overarching categories. The findings will be presented in a narrative format, accompanied by tables and potentially supplemented with graphs or diagrams.

As it is beyond the scope of a scoping review [75], no formal methodological quality assessment of the included studies will be conducted.

## Results

**Figure 1:**
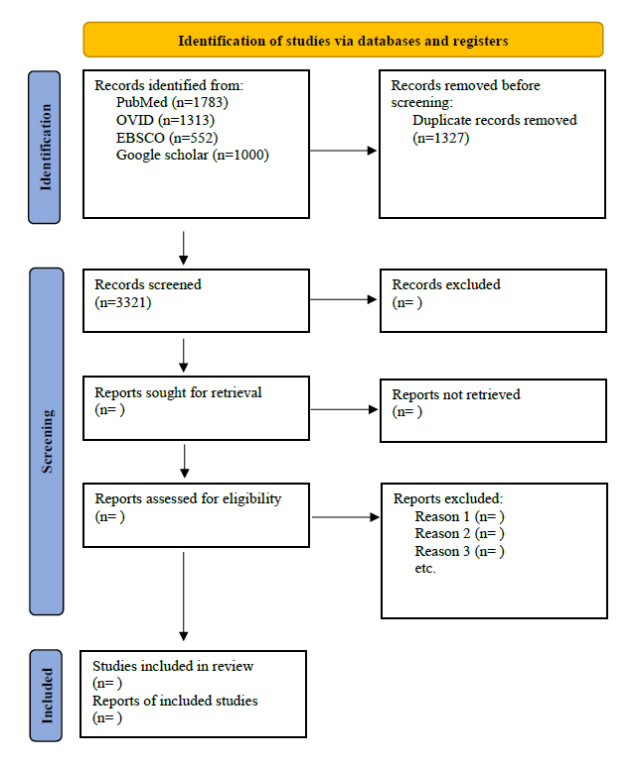
PRISMA 2020 flow diagram for new systematic reviews, which included searches of databases and registers only [73].

Electronic database searches yielded 3321 results after removing duplicates (Figure 1), encompassing articles published between 2014 and August 2024. As of November 2024, screening of titles and abstracts is in progress. To date, no further analysis of the literature has been conducted. The final selection of studies is scheduled to be completed in January 2025, followed by the analysis phase, which is anticipated to conclude in the first quarter of 2025. The results of the study are expected to be published in the second quarter of 2025.

## Discussion

### Principal Findings

This scoping review protocol emphasizes the importance of integrating ethical considerations into empirical eHealth evaluation research. It underscores the need for practical guidance tailored to interdisciplinary teams and aligned with established evaluation practices, including references to study designs, methodologies, and objectives. However, the limited understanding of how ethical considerations are addressed in eHealth evaluation practices presents a significant challenge to achieving this objective. To address this gap, this scoping review will systematically analyze eHealth evaluation studies from the past decade to assess how ethical considerations have been incorporated.

We assume that ethical considerations are addressed more frequently in eHealth evaluation research than previous reviews indicated. This is because the established standards in health care, evaluation, and research mostly reflect ethical norms, as stated by Leiber and Meyer [76] in the context of evaluation practice. These often hidden ethical norms likely play a significant role in promoting the integration of ethical considerations into eHealth evaluation research practices. However, ethical considerations may not always be addressed systematically or clearly presented in the final publications.

Unlike previous reviews that provide a simple yes or no assessment, this scoping review aims to offer a more nuanced understanding of eHealth evaluation practices concerning ethical aspects. Rather than focusing solely on the substantive level, we emphasize the importance of examining the methods used to identify these ethical considerations. Among the existing reviews, the review by Steerling et al [25] stands out as the only one that examines the practices associated with the reported ethical aspects. Their findings revealed a significant methodological shortfall, with 52.9% of the included studies lacking references to ethical frameworks, indicating a gap in systematic ethical approaches in eHealth research.

A common limitation of existing reviews is the focus on explicitly labeled ethical aspects. Articles that addressed ethical concerns only indirectly were excluded. This issue was pointed out by Droste et al [26] in their investigation of HTA reports and by Keenan et al [22] in the discussion of their study’s limitations. These exclusions, coupled with the lack of information on ethical approaches, contribute to a distorted representation of the practices concerning ethical aspects in eHealth and hinder efforts to solve the problem of underrepresentation of ethical considerations in the field.

Whether neglected or addressed implicitly, ethical aspects may remain invisible to readers, which can significantly impact eHealth practice. For instance, ethical concerns might be disregarded in individual eHealth projects informed by evaluation research or overlooked in systematic reviews and HTA reports that guide health care practices and policies. This bears the risk that the value of eHealth interventions will mainly be evaluated based on the empirical evidence. This evidence is important, but without systematic critical reflections on broader questions like, “Do we want these profound changes in healthcare? Are they in line with ethical standards?” professionals cannot ensure that eHealth technologies are ethically sound [15].

Both empirical and ethical perspectives are crucial for assessing the overall value of eHealth and for addressing emerging ethical risks, such as growing health disparities, before they become irreversible [52,77,78]. To ensure that ethical considerations are visible in future research, we support the suggestions of van der Wilt et al [43]: We advocate for more holistic integrated approaches to evaluating eHealth interventions within and beyond the context of cancer and cardiovascular care. This will increase the likelihood that ethical concerns will be considered in critical decision-making processes [48], ultimately strengthening the ethical foundation of eHealth practices.

### Limitations

Our study will face limitations related to its focus and the complexity of ethics. First, the exclusive focus on cancer and cardiovascular diseases stems from anticipating significant growth in these research areas regarding RPM applications [4,79], considering them as examples for less-studied diseases and eHealth solutions. Our exclusive focus limits the generalizability of our findings to broader eHealth evaluation practices. However, narrowing our scope to specific areas is necessary because of the growing volume of results in eHealth evaluation research and our resource constraints.

Second, limitations arise from the broad concept of ethical aspects, posing challenges to identification and extraction. To tackle this, we will conduct pilot extractions and involve at least two reviewers in the data extraction process, increasing the likelihood of capturing relevant ethical aspects.

### Conclusions

Evaluation studies play a crucial role in shaping daily eHealth practices, from software development and health care delivery to public and policy discussions, by providing well-founded insights. This creates a significant responsibility for researchers and evaluators to identify and address (potential) ethical challenges.

However, ethical aspects are often overlooked or only indirectly addressed in eHealth research. This creates a risk that such considerations may not be adequately reflected in critical decision-making about eHealth.

The proposed scoping review is an initial step toward addressing the neglect of ethical aspects in original eHealth evaluation research. It will offer detailed insights into practices related to ethical considerations, highlighting opportunities for their holistic integration. These findings can inform the development of future guidance and recommendations on how to incorporate ethical aspects into original eHealth evaluation research. In this way, this review will support the advancement of more ethical practices in the eHealth context.

## Appendix

Multimedia Appendix 1 PRISMA-ScR fillable checklist.

Multimedia Appendix 2 Full search strategy.

Multimedia Appendix 3 AI - Paperpal edit.

## Abbreviations

CONSORT: Consolidated Standards of Reporting Trials
DARE: new DAta new REsponsibilities
HTA: Health Technology Assessment
JBI: Joanna Briggs Institute
PRISMA: Preferred Reporting Items for Systematic Reviews and Meta-Analyses
PRISMA-ScR: Preferred Reporting Items for Systematic Reviews and Meta-Analyses extension for Scoping Reviews
RPM: remote patient monitoring
VALIDATE: VALues In Doing Assessments of health Technologies
WHO: World Health Organization

## Data Availability

All data generated during this scoping review will be included in this published article.
