# Supplementary material for: Examining Practices Related to Ethical Aspects in eHealth Evaluation Research: Protocol for a Scoping Review
Source: JMIR Res Protoc. 2025 May 5;14:e60849. doi: 10.2196/60849 (PMC12089876; doi:10.2196/60849)
Supplement: Multimedia Appendix 2 [file resprot_v14i1e60849_app2.docx]

This is a Multimedia Appendix to a full manuscript published in the J Med Internet Res. For full copyright and citation information see <http://dx.doi.org/10.2196/jmir.60849>.

## Search Strategies in Databases

## PubMed

The search strategy for MEDLINE via PubMed, conducted on August 15, 2024, yielded 1,783 results.

((MHEALTH[TI] OR "MOBILE HEALTH"[TI] OR EHEALTH[TI] OR TELEMEDICINE[TI] OR "DIGITAL HEALTH"[TI] OR "PATIENT MONITOR*"[TI] OR "DIGITAL MONITOR*"[TI] OR "DISTANT CONTROL"[TI] OR "ELECTRONIC MONITOR*"[TI] OR "REMOTE MONITOR*"[TI] OR "REMOTE CARE"[TI] OR "REMOTE PATIENT MONITOR*"[TI] OR "REMOTE HEALTH MONITOR*"[TI] OR "REMOTE CONTROL"[TI] OR "REMOTE TECHNOLOG*"[TI] OR "HOME MONITOR*"[TI] OR "TELE MONITOR*"[TI] OR TELEMONITOR*[TI] OR "HEALTH MONITOR*"[TI] OR "HEALTH TRACK*"[TI] OR "HEALTH DATA MONITOR*"[TI] OR "BEHAVIOR TRACK*"[TI] OR "BEHAVIOR MONITOR*"[TI] OR "SYMPTOM TRACK*"[TI] OR "SYMPTOM MONITOR*"[TI] OR EPRO[TI] OR "ELECTRONIC PATIENT REPORTED OUTCOME*"[TI] OR "PATIENT-GENERATED OUTCOME*"[TI] OR "ELECTRONIC DATA PROCESS*"[TI] OR "REAL-TIME MONITOR*"[TI] OR "SENSOR TECHNOLOG*"[TI] OR "REMOTE SENSING TECHNOLOGY"[MAJR] OR "TELEMEDICINE"[MAJR]) AND ("EVALUATION DESIGN*"[TIAB] OR "EVALUATION STUD*"[TIAB] OR "QUANTITATIVE RESEARCH"[TIAB] OR "QUALITATIVE RESEARCH"[TIAB] OR "QUALITATIVE ANALYSIS"[TIAB] OR "QUANTITATIVE ANALYSIS"[TIAB] OR "QUALITATIVE STUD*"[TIAB] OR "QUANTITATIVE STUD*"[TIAB] OR "MIXED METHOD*"[TIAB] OR "MIXED-METHOD*"[TIAB] OR "MULTIMETHOD*"[TIAB] OR "MULTI METHOD*"[TIAB] OR "CLINICAL TRIAL*"[TIAB] OR "PILOT STUD*"[TIAB] OR "FEASIBILITY STUD*"[TIAB] OR "IMPLEMENTATION STUD*"[TIAB] OR "IMPLEMENTATION RESEARCH*"[TIAB] OR "RANDOMIZED CONTROLLED TRIAL*"[TIAB] OR "RCT"[TIAB] OR "OBSERVATIONAL STUD*"[TIAB] OR "OUTCOME ASSESSMENT*"[TIAB] OR "PROCESS EVALUATION*"[TIAB] OR "OUTCOME EVALUATION*"[TIAB] OR "PROGRAM EVALUATION*"[TIAB] OR "PROGRAM EVALUATION"[MAJR] OR "EVALUATION STUDIES AS TOPIC"[MAJR])) AND (CARDIOLOGY[TI] OR "CARDIOVASCULAR DISEASE*"[TI] OR "HEART DISEASE*"[TI] OR ATHEROSCLEROSIS[TI] OR HYPERTENSION[TI] OR "HIGH BLOOD PRESSURE"[TI] OR STROKE*[TI] OR "VASCULAR DISEASE*"[TI] OR "CORONARY ARTERY DISEASE*"[TI] OR "MYOCARDIAL INFARCT*"[TI] OR ANGINA[TI] OR "HEART FAILURE*"[TI] OR ARRHYTHM*[TI] OR CARDIOMYOPATHY[TI] OR "ISCHEMIC HEART DISEASE*"[TI] OR "PERIPHERAL ARTERIAL DISEASE*"[TI] OR ANEURYSM[TI] OR "CARDIAC ARREST"[TI] OR "HEART VALVE DISEASE*"[TI] OR "CONGENITAL HEART DISEASE*"[TI] OR "VENOUS THROMBO*"[TI] OR "PULMONARY HYPERTENSION"[TI] OR "CARDIOVASCULAR DISEASES"[MAJR] OR "STEM CELL TRANSPLANT*"[TI] OR "BONE MARROW TRANSPLANT*"[TI] OR "AUTOLOGOUS TRANSPLANT*"[TI] OR "ALLOGENEIC TRANSPLANT*"[TI] OR "BLOOD TRANSPLANT*"[TI] OR "NEOPLASMS"[TI] OR "ADENOMA*"[TI] OR "ANTICARCINOGEN*"[TI] OR "BLASTOMA*"[TI] OR "CANCER*"[TI] OR "CARCINOGEN*"[TI] OR "CARCINOM*"[TI] OR "CARCINOSARCOMA*"[TI] OR "CHORDOMA*"[TI] OR "GERMINOMA*"[TI] OR "GONADOBLASTOMA*"[TI] OR "HEPATOBLASTOMA*"[TI] OR "HODGKIN DISEASE*"[TI] OR "HODGKINS DISEASE*"[TI] OR "LEUKEMI*"[TI] OR "LEUKAEMI*"[TI] OR "LYMPHANGIOMA*"[TI] OR "LYMPHANGIOMYOMA*"[TI] OR "LYMPHANGIOSARCOMA*"[TI] OR "LYMPHOM*"[TI] OR "MALIGNAN*"[TI] OR "MELANOM*"[TI] OR "MENINGIOMA*"[TI] OR "MESENCHYMOMA*"[TI] OR "MESONEPHROMA*"[TI] OR "METASTA*"[TI] OR "NEOPLAS*"[TI] OR "NEUROMA*"[TI] OR "NSCLC"[TI] OR "ONCOGEN*"[TI] OR "ONCOLOG*"[TI] OR "PARANEOPLASTIC"[TI] OR "PLASMACYTOMA*"[TI] OR "PRECANCEROUS"[TI] OR "SARCOMA*"[TI] OR "TERATOCARCINOMA*"[TI] OR "TERATOMA*"[TI] OR "TUMOR*"[TI] OR "TUMOUR*"[TI] OR "NEOPLASMS"[MAJR]) Filters: in the last 10 years, English, German Sort by: Most Recent

## EBSCOhost

The search strategy for CINAHL, SocINDEX with Full Text, and Philosopher's Index via EBSCOhost, conducted on August 15, 2024, yielded 554 results.

( ((TI (CARDIOLOGY OR "CARDIOVASCULAR DISEASE*" OR "HEART DISEASE*" OR ATHEROSCLEROSIS OR HYPERTENSION OR "HIGH BLOOD PRESSURE" OR STROKE* OR "VASCULAR DISEASE*" OR "CORONARY ARTERY DISEASE*" OR "MYOCARDIAL INFARCT*" OR ANGINA OR "HEART FAILURE*" OR ARRHYTHM* OR CARDIOMYOPATHY OR "ISCHEMIC HEART DISEASE*" OR "PERIPHERAL ARTERIAL DISEASE*" OR ANEURYSM OR "CARDIAC ARREST" OR "HEART VALVE DISEASE*" OR "CONGENITAL HEART DISEASE*" OR "VENOUS THROMBOEMBOLISM" OR "PULMONARY HYPERTENSION" OR "STEM CELL TRANSPLANT*" OR "BONE MARROW TRANSPLANT*" OR "AUTOLOGOUS TRANSPLANT*" OR "ALLOGENEIC TRANSPLANT*" OR "BLOOD TRANSPLANT*" OR "NEOPLASMS" OR "ADENOMA*" OR "ANTICARCINOGEN*" OR "BLASTOMA*" OR "CANCER*" OR "CARCINOGEN*" OR "CARCINOM*" OR "CARCINOSARCOMA*" OR "CHORDOMA*" OR "GERMINOMA*" OR "GONADOBLASTOMA*" OR "HEPATOBLASTOMA*" OR "HODGKIN DISEASE*" OR "HODGKINS DISEASE*" OR "LEUKEMI*" OR "LEUKAEMI*" OR "LYMPHANGIOMA*" OR "LYMPHANGIOMYOMA*" OR "LYMPHANGIOSARCOMA*" OR "LYMPHOM*" OR "MALIGNAN*" OR "MELANOM*" OR "MENINGIOMA*" OR "MESENCHYMOMA*" OR "MESONEPHROMA*" OR "METASTA*" OR "NEOPLAS*" OR "NEUROMA*" OR "NSCLC" OR "ONCOGEN*" OR "ONCOLOG*" OR "PARANEOPLASTIC" OR "PLASMACYTOMA*" OR "PRECANCEROUS" OR "SARCOMA*" OR "TERATOCARCINOMA*" OR "TERATOMA*" OR "TUMOR*" OR "TUMOUR*")) OR (MM ("NEOPLASMS" OR "CARDIOVASCULAR DISEASES"))) ) AND ( (TI ('EVALUATION DESIGN*' OR 'EVALUATION STUD*' OR 'QUANTITATIVE RESEARCH' OR 'QUALITATIVE RESEARCH' OR 'QUALITATIVE ANALYSIS' OR 'QUANTITATIVE ANALYSIS' OR 'QUALITATIVE STUD*' OR 'QUANTITATIVE STUD*' OR 'MIXED METHOD*' OR 'MIXED-METHOD*' OR 'MULTIMETHOD*' OR 'MULTI METHOD*' OR 'CLINICAL TRIAL*' OR 'PILOT STUD*' OR 'FEASIBILITY STUD*' OR 'IMPLEMENTATION STUD*' OR 'IMPLEMENTATION RESEARCH*' OR 'RANDOMIZED CONTROLLED TRIAL*' OR 'RCT' OR 'OBSERVATIONAL STUD*' OR 'OUTCOME ASSESSMENT*' OR 'PROCESS EVALUATION*' OR 'OUTCOME EVALUATION*' OR 'PROGRAM EVALUATION*')) OR (AB ('EVALUATION DESIGN*' OR 'EVALUATION STUD*' OR 'QUANTITATIVE RESEARCH' OR 'QUALITATIVE RESEARCH' OR 'QUALITATIVE ANALYSIS' OR 'QUANTITATIVE ANALYSIS' OR 'QUALITATIVE STUD*' OR 'QUANTITATIVE STUD*' OR 'MIXED METHOD*' OR 'MIXED-METHOD*' OR 'MULTIMETHOD*' OR 'MULTI METHOD*' OR 'CLINICAL TRIAL*' OR 'PILOT STUD*' OR 'FEASIBILITY STUD*' OR 'IMPLEMENTATION STUD*' OR 'IMPLEMENTATION RESEARCH*' OR 'RANDOMIZED CONTROLLED TRIAL*' OR 'RCT' OR 'OBSERVATIONAL STUD*' OR 'OUTCOME ASSESSMENT*' OR 'PROCESS EVALUATION*' OR 'OUTCOME EVALUATION*' OR 'PROGRAM EVALUATION*')) OR (MM ("FORMATIVE EVALUATION RESEARCH" OR "SUMMATIVE EVALUATION RESEARCH" OR "EVALUATION RESEARCH" OR "PROSPECTIVE STUDIES" OR "NONEXPERIMENTAL STUDIES" OR "VALIDATION STUDIES" OR "EXPERIMENTAL STUDIES" OR "MULTIMETHOD STUDIES" OR "PILOT STUDIES" OR "Quantitative Studies" OR "Multicenter Studies")) ) AND ( ((TI (MHEALTH OR "MOBILE HEALTH" OR "EHEALTH" OR "TELEMEDICINE" OR "DIGITAL HEALTH" OR "PATIENT MONITOR*" OR "DIGITAL MONITOR*" OR "DISTANT CONTROL" OR "ELECTRONIC MONITOR*" OR "REMOTE MONITOR*" OR "REMOTE CARE" OR "REMOTE PATIENT MONITOR*" OR "REMOTE HEALTH MONITOR*" OR "REMOTE CONTROL" OR "REMOTE TECHNOLOG*" OR "HOME MONITOR*" OR "TELE MONITOR*" OR "TELEMONITOR*" OR "HEALTH MONITOR*" OR "HEALTH TRACK*" OR "HEALTH DATA MONITOR*" OR "BEHAVIOR TRACK*" OR "BEHAVIOR MONITOR*" OR "SYMPTOM TRACK*" OR "SYMPTOM MONITOR*" OR EPRO OR "ELECTRONIC PATIENT REPORTED OUTCOME*" OR "PATIENT-GENERATED OUTCOME*" OR "ELECTRONIC DATA PROCESS*" OR "REAL-TIME MONITOR*" OR "SENSOR TECHNOLOG*" OR "REMOTE SENSING TECHNOLOGY")) OR (MM "MONITORING, PHYSIOLOGIC")) ) Filter 2024-2024

## OVID

The search strategy for Embase and PsycINFO via Ovid, conducted on August 15, 2024, yielded 1,313 results.

(('EVALUATION DESIGN*' OR 'EVALUATION STUD*' OR 'QUANTITATIVE RESEARCH' OR 'QUALITATIVE RESEARCH' OR 'QUALITATIVE ANALYSIS' OR 'QUANTITATIVE ANALYSIS' OR 'QUALITATIVE STUD*' OR 'QUANTITATIVE STUD*' OR 'MIXED METHOD*' OR 'MIXED-METHOD*' OR 'MULTIMETHOD*' OR 'MULTI METHOD*' OR 'CLINICAL TRIAL*' OR 'PILOT STUD*' OR 'FEASIBILITY STUD*' OR 'IMPLEMENTATION STUD*' OR 'IMPLEMENTATION RESEARCH*' OR 'RANDOMIZED CONTROLLED TRIAL*' OR 'RCT' OR 'OBSERVATIONAL STUD*' OR 'OUTCOME ASSESSMENT*' OR 'PROCESS EVALUATION*' OR 'OUTCOME EVALUATION*' OR 'PROGRAM EVALUATION*').TI,AB. OR *EVALUATION RESEARCH/ OR *EVALUATION STUDY/ OR *"EVALUATION AND FOLLOW UP"/ OR *PROGRAM EVALUATION/) AND ((MHEALTH OR "MOBILE HEALTH" OR "EHEALTH" OR "TELEMEDICINE" OR "DIGITAL HEALTH" OR "PATIENT MONITOR*" OR "DIGITAL MONITOR*" OR "DISTANT CONTROL" OR "ELECTRONIC MONITOR*" OR "REMOTE MONITOR*" OR "REMOTE CARE" OR "REMOTE PATIENT MONITOR*" OR "REMOTE HEALTH MONITOR*" OR "REMOTE CONTROL" OR "REMOTE TECHNOLOG*" OR "HOME MONITOR*" OR "TELE MONITOR*" OR "TELEMONITOR*" OR "HEALTH MONITOR*" OR "HEALTH TRACK*" OR "HEALTH DATA MONITOR*" OR "BEHAVIOR TRACK*" OR "BEHAVIOR MONITOR*" OR "SYMPTOM TRACK*" OR "SYMPTOM MONITOR*" OR EPRO OR "ELECTRONIC PATIENT REPORTED OUTCOME*" OR "PATIENT-GENERATED OUTCOME*" OR "ELECTRONIC DATA PROCESS*" OR "REAL-TIME MONITOR*" OR "SENSOR TECHNOLOG*" OR "REMOTE SENSING TECHNOLOGY").TI. OR *TELEMONITORING/) AND ((CARDIOLOGY OR "CARDIOVASCULAR DISEASE*" OR "HEART DISEASE*" OR ATHEROSCLEROSIS OR HYPERTENSION OR "HIGH BLOOD PRESSURE" OR STROKE* OR "VASCULAR DISEASE*" OR "CORONARY ARTERY DISEASE*" OR "MYOCARDIAL INFARCT*" OR ANGINA OR "HEART FAILURE*" OR ARRHYTHM* OR CARDIOMYOPATHY OR "ISCHEMIC HEART DISEASE*" OR "PERIPHERAL ARTERIAL DISEASE*" OR ANEURYSM OR "CARDIAC ARREST" OR "HEART VALVE DISEASE*" OR "CONGENITAL HEART DISEASE*" OR "VENOUS THROMBOEMBOLISM" OR "PULMONARY HYPERTENSION" OR "STEM CELL TRANSPLANT*" OR "BONE MARROW TRANSPLANT*" OR "AUTOLOGOUS TRANSPLANT*" OR "ALLOGENEIC TRANSPLANT*" OR "BLOOD TRANSPLANT*" OR "NEOPLASMS" OR "ADENOMA*" OR "ANTICARCINOGEN*" OR "BLASTOMA*" OR "CANCER*" OR "CARCINOGEN*" OR "CARCINOM*" OR "CARCINOSARCOMA*" OR "CHORDOMA*" OR "GERMINOMA*" OR "GONADOBLASTOMA*" OR "HEPATOBLASTOMA*" OR "HODGKIN DISEASE*" OR "HODGKINS DISEASE*" OR "LEUKEMI*" OR "LEUKAEMI*" OR "LYMPHANGIOMA*" OR "LYMPHANGIOMYOMA*" OR "LYMPHANGIOSARCOMA*" OR "LYMPHOM*" OR "MALIGNAN*" OR "MELANOM*" OR "MENINGIOMA*" OR "MESENCHYMOMA*" OR "MESONEPHROMA*" OR "METASTA*" OR "NEOPLAS*" OR "NEUROMA*" OR "NSCLC" OR "ONCOGEN*" OR "ONCOLOG*" OR "PARANEOPLASTIC" OR "PLASMACYTOMA*" OR "PRECANCEROUS" OR "SARCOMA*" OR "TERATOCARCINOMA*" OR "TERATOMA*" OR "TUMOR*" OR "TUMOUR*").TI. OR *NEOPLASMS/ OR *MALIGNANT NEOPLASM/ OR *CARDIOVASCULAR DISEASE/ OR *CARDIOLOGY/ OR *CARDIOVASCULAR DISORDERS/) Filter: 2014-2024

## Google Scholar

The search strategy for Google Scholar using Publish or Perish was conducted on August 16, 2024. The first 1,000 results were screened.

("eHealth" OR "mHealth" OR "remote monitoring" OR "patient monitor*" OR "electronic patient reported outcomes") AND ("cardiology" OR "cardiovascular disease*" OR "heart disease*" OR "cancer" OR "oncology" OR "neoplasm*" OR "tumor*") AND ("evaluation study" OR "evaluation research") Filter: 2014-2024
