# Supplementary material for: Examining Practices Related to Ethical Aspects in eHealth Evaluation Research: Protocol for a Scoping Review
Source: JMIR Res Protoc. 2025 May 5;14:e60849. doi: 10.2196/60849 (PMC12089876; doi:10.2196/60849)
Supplement: Multimedia Appendix 3 [file resprot_v14i1e60849_app3.docx]

| 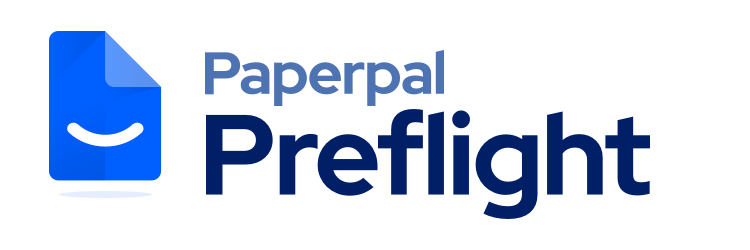 | Paperpal Manuscript Processing Report  Date: Dec 11, 2024 |
| --- | --- |
| Abstract is too long | The abstract is longer than recommended. It should be between 0 and 200 words. |
| [Missing author list](#author_compliance) | It looks like the manuscript does not include the list of authors. It is important that all authors who have contributed to the paper are credited. |
| Missing author's email | It looks like the manuscript is missing an email address for the corresponding author. It is important to include this email address as the corresponding author is the point of contact for editors as well as readers. |
| Manuscript is too long | The manuscript is longer than recommended. It should be between 2,500 and 4,000 words. |
| Manuscript does not follow the IMRaD structure | It looks like the manuscript doesn't follow the IMRaD ( [Wikipedia Definition](https://en.wikipedia.org/wiki/IMRAD) ) structure. It is recommended that the manuscript follows the IMRaD to ensure that the research is conveyed in an effective manner. |
| Missing plain language summary | It looks like the manuscript is missing a plain language summary. |
| [Missing word count](#word_counts) | It looks like the manuscript does not include a word count. It is recommended to report the word count of the manuscript. |
| Issues found in Language quality - readability | Readability suggestions covers issues like conciseness, redundancy, transition and flow, and suggests improved phrasing. We have identified 106 issues in this category. |

**Review Protocol**

## Examining Practices Related to Ethical Aspects in eHealth Evaluation Research: Protocol for a Scoping Review

Jane AUTEUR, Institute of Published Science, University of Examples, Singapore, and John WRIGHT, Institute of Submissions, School of Technicalities, USA.

## Abstract

**Background:** eHealth technologies, including remote patient monitoring (RPM) applications, have the potential to improve care for diseases, such as cancer and cardiovascular conditions. However, they also raise ethical concerns that are often inadequately addressed in eHealth evaluation research. This is problematic, as evaluations guide decision making at multiple levels. To improve evaluation practices, it is essential to understand how ethical aspects are addressed in terms of both content and methodology, enabling the development of tailored recommendations for enhancement.

**Objective:** This scoping review systematically examines how ethical aspects are addressed in eHealth research, focusing on original studies evaluating RPM applications for cancer and cardiovascular diseases.

**Methods:** Using JBI methodology and PRISMA-ScR guidelines, this review implemented a comprehensive search strategy with the terms "cancer or cardiovascular diseases," "eHealth or telemonitoring," and "evaluation designs." Searches included MEDLINE, Embase, CINAHL, SocINDEX, Philosophers Index, PsychINFO, and Google Scholar. Data extraction emphasizes ethical aspects and methodological approaches to consider them. The analysis applies inductive-deductive qualitative content analysis.

**Results:** The initial searches identified 3,321 articles published between 2014 and August 2024. Screening and analysis will be completed in the first quarter of 2025, with results anticipated by the spring of 2025.

**Conclusions:** Incorporating ethical considerations into evaluation research can enhance its influence on critical decision-making processes. This scoping review aims to develop practical guidance for interdisciplinary teams, enabling them to systematically integrate these considerations into their work, thereby promoting ethical eHealth practices.

**Protocol Registration:** This protocol is registered on the Open Science Framework website (registration link: https://osf.io/7xafv/).

Word count: 8,067 words, excluding references.

**Keywords:** cancer; cardiovascular; eHealth; remote patient monitoring; ethics; ethical aspects; evaluation designs; evaluation research
